# Multiple Distal Femoral Osteochondromas Encasing Popliteal Neurovascular Bundle

**DOI:** 10.7759/cureus.46396

**Published:** 2023-10-03

**Authors:** Amir Habeeb, Jaroszlav Roszpopa, Ali Arnaout, Ian Grant, Mark Latimer

**Affiliations:** 1 Otolaryngology, Cambridge University Hospitals NHS Foundation Trust, Cambridge, GBR; 2 Plastic Surgery, Cambridge University Hospitals NHS Foundation Trust, Cambridge, GBR; 3 Orthopedics, University of Leicester Medical School, Leicester, GBR

**Keywords:** neurovascular conservation, cancer-specific outcome, surgical case reports, soft tissue tumour, orthopaedics surgery

## Abstract

Multiple hereditary exostosis syndrome is a rare diagnosis with approximately 1:50000 incidence prevailing in males. The exostoses or osteochondromas are benign but have the potential for malignant transformation in 1-5%. There is a strong genetic component, with exostosis (EXT) signaling pathways being an underlying cause. They can be symptomatic, with pain and functional deficit as the main complaints. We present a case of a 17-year-old male who presented with pain and anatomical deformity in his left lower femur. Magnetic resonance imaging revealed multiple osteochondromas compressing the popliteal neurovascular bundle. Excision of the osteochondromas was performed to decompress the neurovascular bundle in a multidisciplinary approach. Histological examination demonstrated no evidence of malignancy. Currently, there is no consensus for patients diagnosed with multiple osteochondromas regarding further investigation and/or screening for malignant transformation.

## Introduction

Osteochondromas are defined as bony outgrowths with a cartilage cap [1]. They tend to be of long-bone origin [1]. Multiple lesions have a prevalence of roughly 1:50000 [2], with an increased likelihood of occurring in males at 1.5:1 [3]. They usually present in the first decade and cease to grow when skeletal growth is complete [3]. The numbers and locations can vary between and within families, with the majority being symptomatic [3,4]. In some patients, osteochondromas may cause pain, deformities, and impaired function [4]. In 0.5-5% of osteochondromas, there is malignant transformation potential, hence the need for careful surveillance [2].

Mutations affecting the EXT (exostosis) gene or the repeated presence of a family history of EXT gene abnormality have been reported in the majority of patients [2]. Both EXT 1 and EXT2 mutations are documented, with EXT1 more likely to cause family inheritance patterns whilst EXT2 is associated with random mutations [5,6]. These genes are tumor suppressor genes, and the cartilage cap of osteochondromas has been solely shown to have a loss of the EXT1 wildtype allele, emphasizing the neoplastic nature of the cartilage cap whilst the stalk is a reactive process [5,6].

Exostosin-1 and 2 are the gene products - they are endoplasmic reticulum localized type II transmembrane glycoproteins crucial in forming a complex that catalyzes a reaction that is critical for heparan sulfate polymerization, an essential step in bone remodeling [7,8]. In cases where EXT decreases, the heparan sulfate proteoglycans accumulate in cell cytoplasms, thus upsetting the careful balance of paracrine signaling that controls bone growth [7,8].

## Case presentation

**Figure 1:**
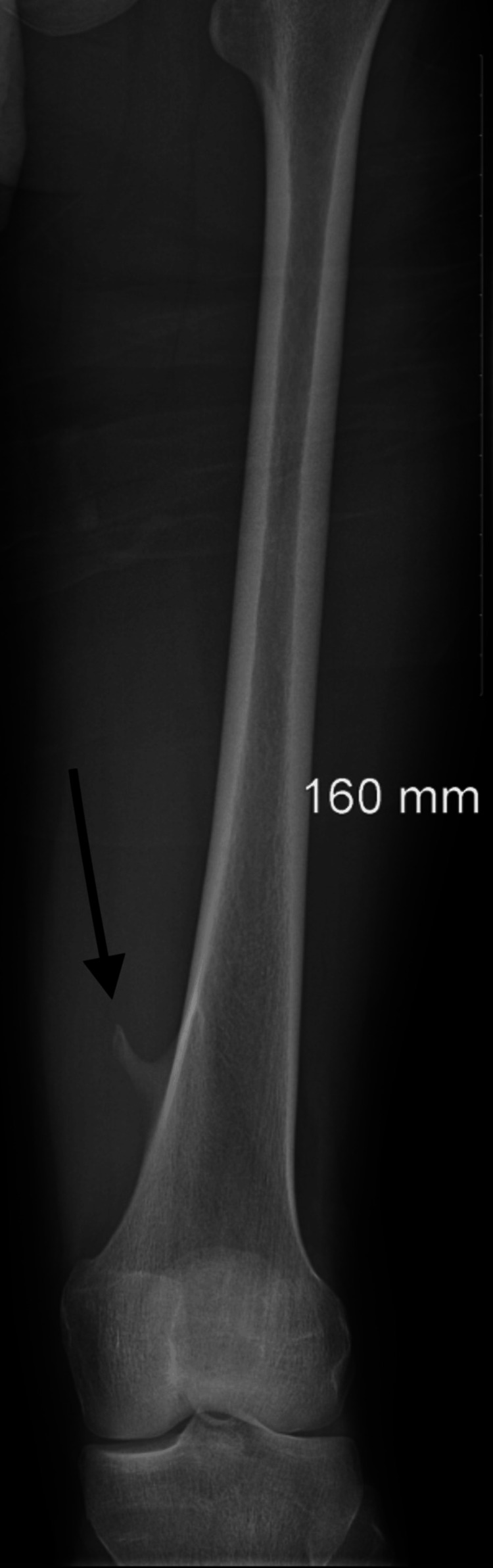
X-ray showing osteochondromas on the left distal femur posteromedially

**Figure 2:**
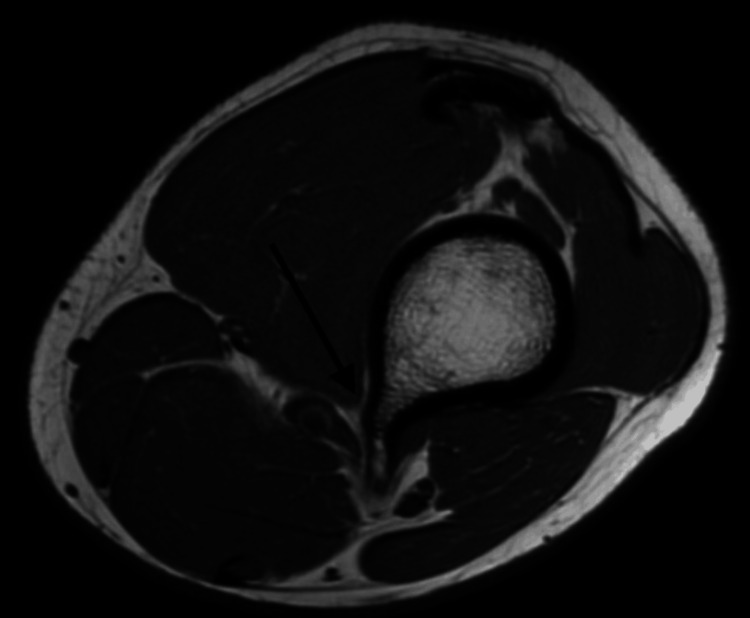
MRI T1-weighted axial imaging showing osteochondromas and their relation to the femur and neurovascular structures

**Figure 3:**
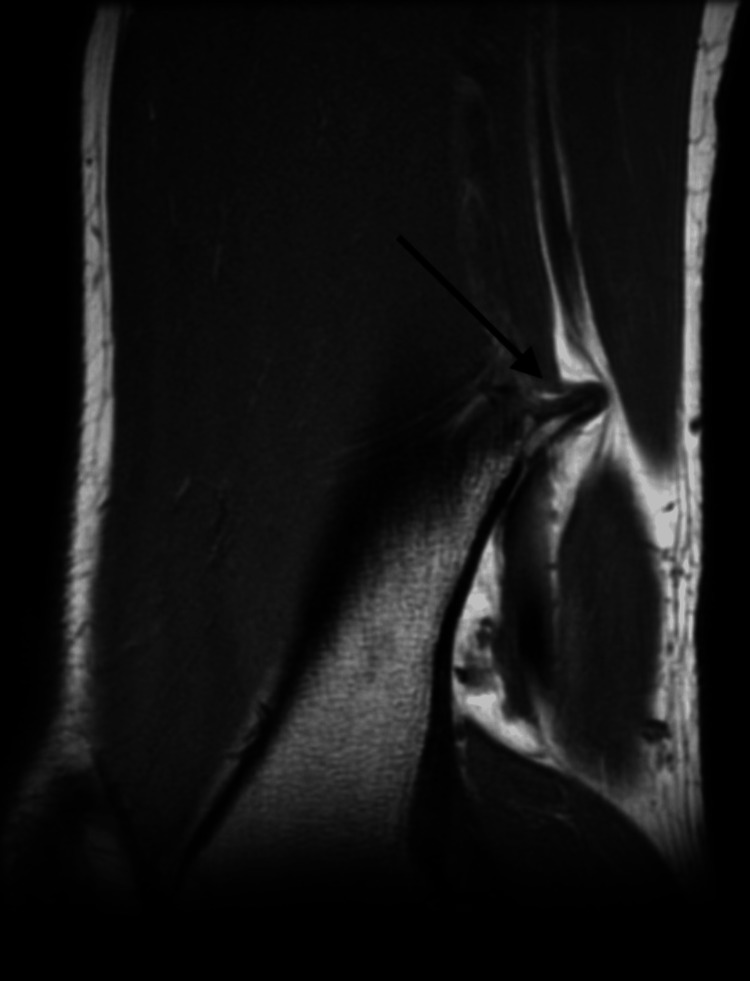
MRI T1-weighted sagittal imaging showing osteochondromas and their relation to the femur and neurovascular structures

A 17-year-old male presented with a one-year history of a painful lump on the distal left thigh, located medial and just above the knee joint, most noticeable on walking. It was becoming progressively worse and more recently limiting the ability to walk and participate in sports. Plain X-ray and MRI were obtained (Figures 1-3). Imaging revealed the presence of two pedunculated osteochondromas arising from the posteromedial distal femoral metaphysis, intimately related to the popliteal neurovascular bundle passing between the bifurcation of the distal sciatic nerve (lateral to the osteochondroma) and the popliteal artery and vein on the medial side. There were no ontologically concerning features. Following a review by the sarcoma clinic, the patient was referred for operative management with the orthopedic team.

**Figure 4:**
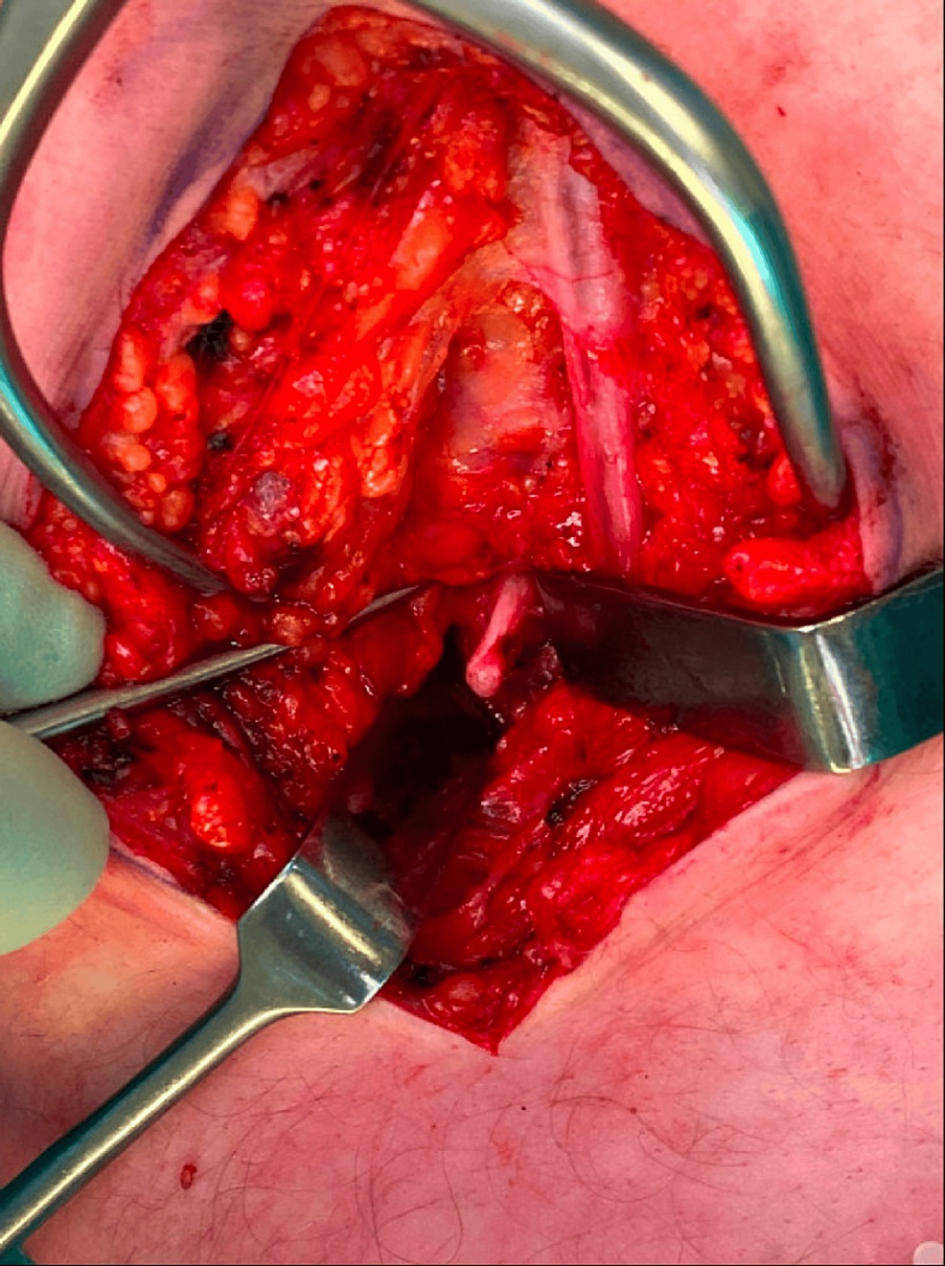
Intraoperative dissection showing proximity to neurovascular structures

**Figure 5:**
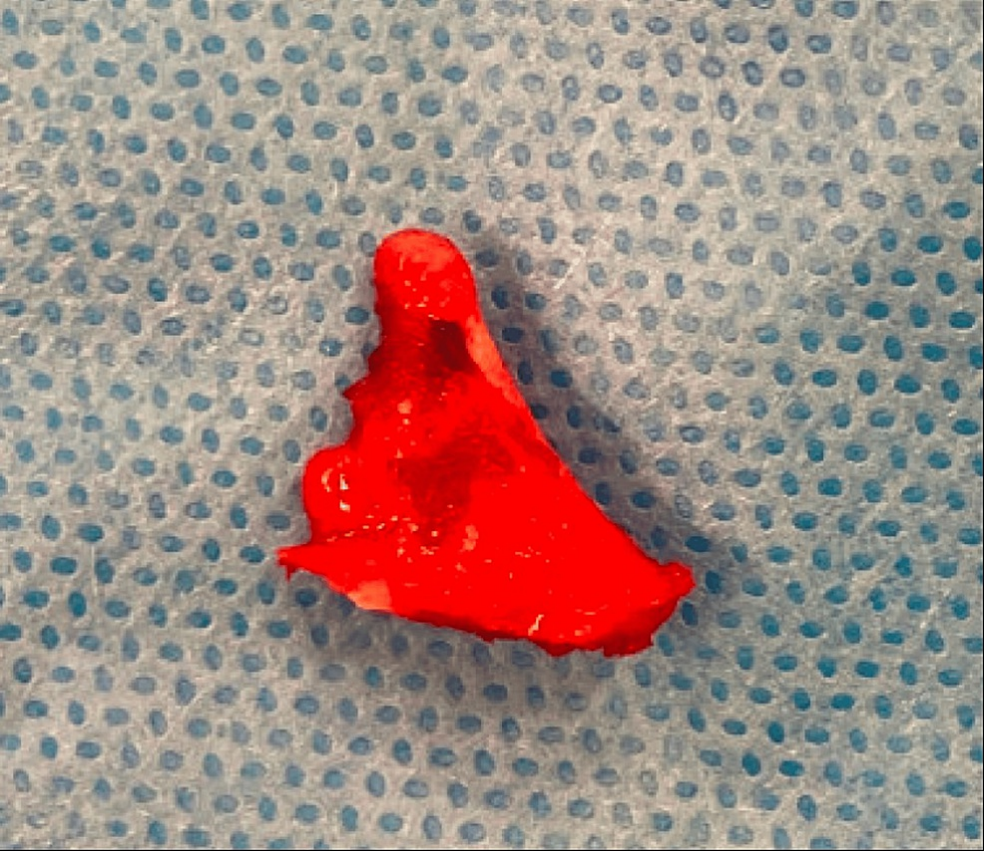
Excised osteochondromas sent for histology

Under general anesthesia, with both orthopedic and plastic surgeons in the team, a combined approach was utilized to excise both osteochondromas (Figure 4 and Figure 5) using a popliteal approach, the neurovascular bundle (sciatic nerve and popliteal artery and vein) was identified and protected. The osteochondromas then were carefully resected to decompress the bundle. The patient was advised to fully weight bear as tolerated. At the six-week follow-up. the patient reported an uneventful recovery and full resolution of his symptoms.

Histology revealed three bony fragments macroscopically together measuring 25 x 15 x 5 mm. Microscopically sections of cortical bone were identified, with one fragment containing a surface cartilaginous tumor. The base of the tumor was sealed by endochondral ossification, with active bone remodeling. The lesion was continuous with the medullary cavity of the underlying bone. The cartilage cap showed no significant cytologic atypia and measured up to 1.5 mm in thickness. There was no evidence of malignancy.

## Discussion

Having multiple osteochondromas is a rare presentation that requires careful understanding to ensure the best outcomes for the patient. Such cases are best managed by a multidisciplinary team that consists of specialists within the field of bone tumors. Given the germline mutations that are most likely the culprits, these patients should be screened for EXT1 and EXT2 mutations. Where a positive family history is present then this screening is not necessary [9,10].

Malignant transformation should be investigated for growth occurring after growth plate fusion using T2-weighted magnetic resonance imaging [11]. This can be done by looking at the cartilage cap where >1.5 cm should raise suspicions [11].

It is important to consider differential diagnoses such as dysplasia epiphysealis hemimelica and metachondromatosis when dealing with solitary and hereditary osteochondromas; however, these have been shown not to impact the EXT pathway [6,7]. Both are rare disorders with the former not being associated with malignant transformation and usually occurring earlier in adolescence, which is in contrast to multiple osteochondromas where malignant transformation is a possibility and they tend to occur in older adolescents [12]. The latter is also important to distinguish as metachondromatosis does not cause limb shortening or deformity of the affected bones and may even spontaneously resolve which is not the case in multiple osteochondromas [13]. Genetic counseling is a vital consideration, as having multiple osteochondromas is an autosomal dominant disorder with nearly 100% penetrance [14].

Osteochondromas are benign lesions where the risk of malignant transformation is 1-5% The decision to excise them is typically dictated by the symptoms the patient may be experiencing, which are usually pain and functional complaints of nerve/vessel compression [14,15]. Complete excision of multiple osteochondromas should always be considered, which can correct the associated deformity [15]. As no new osteochondromas develop after growth plate fusion, a baseline bone scan is recommended to allow comparison when screening although there are no well-documented screening frequencies or whether this is even of benefit [9].

In case of malignancy or proximity to important structures, careful dissection is advised as well as en-bloc resection of the osteochondromas and its pseudocapsule to ensure tumor-free margins [16].

## Conclusions

Having multiple osteochondromas is a rare and complex diagnosis that is still yet to be properly understood. Mainstay management includes history, examination, and thorough radiological assessment. The main presenting complaints include pain and a functional deficit. They are associated commonly with a mutation of the EXT gene and should be managed by a specialist multidisciplinary team to achieve a favorable outcome by surgical intervention to reduce the burden of symptoms. Signs and symptoms suggestive of malignant transformation include relapsing and/or worsening pain, regrowth of the tumor, as well as histological features such as internal lytic areas, irregular margins or calcifications, and a cartilage cap thickness >2 cm in adults or >3 cm in children. A screening protocol following primary surgery may offer benefits in the long term in identifying at-risk patients.
